# Repurposing Waste from Aggressive *Acacia* Invaders to Promote Its Management in Large Invaded Areas in Southwestern Europe

**DOI:** 10.3390/plants13111428

**Published:** 2024-05-21

**Authors:** Paula Lorenzo, Maria Cristina Morais

**Affiliations:** 1 Centre for Functional Ecology (CFE)—Science for People & the Planet, Associate Laboratory TERRA, Department of Life Sciences, University of Coimbra, 3000-456 Coimbra, Portugal; 2 Centre for the Research and Technology of Agro-Environmental and Biological Sciences (CITAB), Inov4Agro, Institute for Innovation, Capacity Building and Sustainability of Agro-Food Production, University of Trás-of-Montes and Alto Douro, Quinta de Prados, 5000-801 Vila Real, Portugal; cmorais@utad.pt

**Keywords:** agricultural and forestry systems, bioherbicides, biostimulants, organic fertilizers, green manure

## Abstract

Several *Acacia* species are aggressive invaders outside their native range, often occupying extensive areas. Traditional management approaches have proven to be ineffective and economically unfeasible, especially when dealing with large infestations. Here, we explain a different approach to complement traditional management by using the waste from *Acacia* management activities. This approach can provide stakeholders with tools to potentially reduce management costs and encourage proactive management actions. It also prioritizes potential applications of *Acacia* waste biomass for agriculture and forestry as a way of sequestering the carbon released during control actions. We advocate the use of compost/vermicompost, green manure and charcoal produced from *Acacia* waste, as several studies have shown their effectiveness in improving soil fertility and supporting crop growth. The use of waste and derivatives as bioherbicides or biostimulants is pending validation under field conditions. Although invasive *Acacia* spp. are banned from commercialization and cultivation, the use of their waste remains permissible. In this respect, we recommend the collection of *Acacia* waste during the vegetative stage and its subsequent use after being dried or when dead, to prevent further propagation. Moreover, it is crucial to establish a legal framework to mitigate potential risks associated with the handling and disposal of *Acacia* waste.

## 1. Introduction

Australian *Acacia* species are aggressive invasive species that severely affect ecosystem structure and functioning globally [1,2]. Recognized as ecosystem transformers [3], invasive *Acacia* species provide some beneficial goods, but adverse impacts tend to outweigh the benefits as their invasion increases [2]. The negative impacts encompass both below- and above-ground components of the ecosystem they invade [4,5].

Some invasive *Acacia* species are widely distributed, covering large areas and forming dense and homogeneous stands [6,7]. In Europe, the invasion of *Acacia* species is particularly severe in the Iberian Peninsula [6,7] and Italy [8,9]. To prevent the spread of *Acacia* propagules in the Iberian Peninsula, for example, the cultivation and commercialization of the most aggressive specimens is prohibited by legislation (in Portugal by the Decree-Law no. 92/10 July 2019; and in Spain by the Royal Decree 630/2 August 2013). In addition, increasing efforts have been made in recent decades to control and prevent introduction of *Acacia* species and increase public and stakeholder engagement [10]. Despite this, the management of invasive *Acacia* species is far from successful, mainly due to the lack of resources to implement long-term control actions in large invaded areas [7,8,9,10,11]. At this point, eradication of aggressive *Acacia* species is considered economically and realistic unfeasible and their management remains challenging [7,11], emphasizing the need to prioritize control in small areas of high ecological value [10].

Some traits of invasive *Acacia* species, including nitrogen-fixation, large quantities of long-lived seeds, rapid growth and vigorous resprouting from stumps or roots following disturbance, contribute to a continuous production of biomass and make its management very difficult [2,10,12,13,14]. This situation, together with a lack of resources, frequently leads stakeholders to abandon *Acacia* control, thereby aggravating the problems of *Acacia* invasion [personal observation]. Here, we advocate the need to overcome these management barriers to avoid abandonment and make control more attractive. The valorization of *Acacia* waste can be a complementary way to potentially reduce costs and stimulate management actions [7,10,11]. There are several potential uses for waste derived from *Acacia* species, which have recently been reviewed by Lorenzo and Morais [11] and López-Hortas [15]. However, there is a knowledge gap regarding what uses should be prioritized. This work aims to extend the findings of Lorenzo and Morais [11] and López-Hortas [15] by identifying uses related to agricultural and forestry purposes, as these systems can act as a sink for high amounts of organic matter, thereby sequestering carbon. We focus on the most promising uses that have been reported for the waste of the most invasive *Acacia* species [16,17], highlighting characteristics that are desirable for agriculture and forestry and discussing their feasible application. We also provide some recommendations for best practices in the use of *Acacia* waste.

## 2. Bioactive Compounds as Potential Bioherbicides or Biostimulants

Chemical compounds from *Acacia* waste that show bioactivity can be a source of natural inhibitors or stimulants of plant growth (Table 1), thereby reducing the application of synthetic agrochemicals responsible for health and environmental concerns.

Studies of *Acacia dealbata* Link waste found that methyl cinnamate, identified in methanolic extracts from flowers, negatively affected early seedling growth and related germination enzymatic activities in species with small seeds such as *Lolium rigidum* Gaudin (weed) and lettuce (crop), whereas it did not affect wheat (crop with larger seeds) under laboratory conditions [18]. Interestingly, concentrations of methyl cinnamate that had a negative effect on *L. rigidum* did not affect wheat, and could help to control this weed in wheat crops [18]. Similarly, water-soluble compounds present in ethanolic extracts obtained from *A. dealbata* leaves consistently reduced the radicle length of *L. sativa* in Petri-dish bioassays with either dimethyl sulfoxide or water, suggesting a potentially strong bioherbicidal activity regardless of assay conditions [19]. Additionally, aqueous extracts from *A. dealbata* bark inhibited the radicle length of several urban weeds in Petri dish bioassays [20], but the negative effect almost disappeared in the presence of field soil [20]. These extracts also tended to reduce plant biomass when sprayed on weed leaves in a greenhouse experiment [20]. Conversely, bark compounds extracted with a methanolic-distilled water solution (97:3 *v*/*v*) increased total biomass and content of soluble sugars of onions grown in salinized agricultural soils [21].

On the other hand, extracts from *Acacia melanoxylon* R.Br. (flowers), *Acacia saligna* (Labill.) H.L. Wendl. (flowers), *Acacia mearnsii* De Wild. (leaves and roots) and *Acacia retinodes* (Schltdl.) (flowers) inhibited germination or growth of different weeds [22].

Both negative and positive effects of compounds/extracts of *Acacia* waste were mainly assessed under controlled conditions, suggesting their potential use as bioherbicides and/or growth promoters. However, these assessments represent only half of the process. We encourage the completion of the process by conducting field studies and identifying chemical composition (not strictly required) to validate the obtained results and better understand and/or forecast the effect of the extracts.

In addition, green manure of *A. dealbata* and *Acacia longifolia* (Andrews) Willd. (leaves and small branches directly incorporated into agricultural soil) was effective in reducing the density of maize-accompanying dicotyledon weeds without significantly affecting crop and soil microbes in pots [23]. At field scale, green manure and mulch of *A. dealbata* partially controlled dicotyledon weeds at sites with low weed density [23]. The phytotoxic effect of *A. dealbata* green manure on weeds was later corroborated by a decrease in weed biomass for three months after green manure incorporation in fields devoted to maize crops, which could contribute to reducing the application of synthetic herbicides in maize-based cropping systems [24]. Despite these interesting results, we consider that the bioherbicidal effect should be proved for crops other than maize.

## 3. Biomass Waste as a Source of Soil Amendments and Organic Fertilizers

Invasive *Acacia* species increase soil nitrogen (N) pools and fluxes through N fixation and they alter the concentrations of other nutrients [2], allowing them to produce abundant plant material and, in turn, copious amounts of litter enriched with N and carbon (C) [25]. The C/N ratio for *A. dealbata* and *A. longifolia* ranged from 13.6 to 30 for the foliar and branch mass fraction [26]. Green waste with a C/N ratio between 20 and 30 is suitable for composting [27]. Therefore, waste from invasive *Acacia* species might provide organic products with fertilizing properties.

Composting of *Acacia* waste, alone or mixed with other organic material, is the most common approach to processing large quantities of plant material and evaluating its use (Table 1). For example, Adam et al. [28] amended field soils with compost generated from the biowaste of *Acacia podalyriifolia* A. Cunn. ex G. Don (woody *A. podalyriifolia* + invasive herbaceous biomass, 1:1 biomass ratio) and compared its effect on plant performance with a commercial compost and soils with no amendment. Nitrogen and phosphorus (P) levels were lower in the biowaste compost than in the commercial one, but the C/N ratio was similar. Despite this, biomass production of 28-day-old maize and pea seedlings was equivalent between biowaste compost and commercial compost and higher than in soils with no amendment. Brito et al. [29] evaluated the procedure feasibility of composting *A. longifolia* and *Acacia melanoxylon* R.Br. waste from control actions. They found that the production of *Acacia* compost (60% *A. longifolia*:40% *A. melanoxylon*) required an extended period and specific technical procedures. However, composting proved to be an effective process for recovering organic matter and nutrients from *Acacia* waste as it rendered the final product suitable for organic soil amendment or replacing pine bark compost in horticultural substrates [29,30]. Composting *Acacia* waste biomass combined with pine bark also provides compost that can be used as a component in horticultural substrates and soil amendments [31]. Similarly, Ulm et al. [32] demonstrated that a mixture of *A. longifolia* waste compost and municipal compost (1:2 *v*/*v*) produced reasonable maize yields (up to 8.3 t ha^−1^) in plots in an urban agricultural context, highlighting the role of this soil amendment in recycling organic waste and increasing soil organic matter, thus mitigating greenhouse gas emissions. In another example, exploring the composting of sewage sludge with *A. dealbata* biomass in a 1:1 ratio resulted in a decline in initial content of heavy metals, and, when added to agricultural soils, this mixture did not negatively affect enzymatic activities related to soil nutrient cycling [33]. Alternatively, vermicompost and charcoal can be good soil amendments. Vermicomposting of *A. dealbata* leaves, flowers and young branches rendered a product without phytotoxicity that met EU quality standards for organic fertilizers [34]. Charcoal produced from *Acacia decurrens* (J.C.Wendl.) Willd. wood resulted in improvements in soil organic carbon and total N and nutrients in soils under the *Eragrostis tef* Zucc-*Acacia decurrens*–charcoal production rotation system, compared with the *Eragrostis tef* monoculture system [35].

However, some growth limitations may also occur when using composts from invasive *Acacia* species. These limitations could be overcome by optimizing some parameters in the composts. For example, compost made with freshly shredded branches of *Acacia cyclops* A. Cunn. ex G. Don. produced vigorous but smaller plants compared with a standard substrate [36,37]. In this case, the quality of seedlings might be increased by improving the initial structure and adjusting drying–wetting and fertilization regimes in the compost. In addition, Bakry et al. [36,37] indicated that such *Acacia*-compost-based growing substrates are of reproducible quality and suitable for the production of forest seedlings in a nursery system, especially in resource-limited countries, due to a simplified and easy-to-monitor production process. In another case, compost or vermicompost from mixtures of 20% *Acacia mearnsii* De Wild bark bagasse and 80% bovine manures showed toxic effects on germination and root elongation of lettuce [38]. However, increasing the proportion of bark bagasse in the mixture by up to 50% reduced the toxicity to safe levels and showed adequate physical and mineral composition for growing acid pH- and Cu-tolerant crops [38].

*Acacia* waste can also be used without being subjected to a composting process, which may facilitate waste management. Lorenzo et al. [24] evaluated the value of *A. dealbata* green manure as an organic fertilizer in maize crops under field conditions. They found that green manure incorporated into the soil four months before sowing can provide some of the nutrients needed for maize growth, with additional N fertilization being necessary to correct minor nutrient deficiencies. This suggests that inorganic fertilization can be partially replaced by *A. dealbata* green manure and that *A. dealbata* waste can be used in agriculture with minimal transformation.

Interestingly, we highlight that most of the studies described in this section were conducted with field soil or under field conditions, mimicking natural conditions and revealing the feasibility of these uses. A very promising study on the use of *Acacia* waste is being conducted within the R3forest project [10]. In this project, compost made from *Acacia* waste is being used in an ongoing reforestation context in Portugal to assess the survival of two native forest species planted in cleared invaded areas. Results from the first year showed that the survival rates of both species more than doubled in the compost treatments compared with the control, suggesting that the benefits of compost might help to counterbalance initial costs of *Acacia* management [10]. The authors of this project also developed a toolbox for modelling *A. longifolia* biomass [26]. With that, stakeholders can easily obtain information on the quantity and quality of *A. longifolia* biomass, which can be used for different purposes [26]. Certainly, this model could be extrapolated to other invasive *Acacia* species with similar growth patterns.

**Table 1:** Summary of possible uses for *Acacia* waste related to agricultural and forestry purposes.

Waste Characteristics	Invasive Species	Geographic Study Area	Material	Proposed Use	Reference
Bioactivity	*Acacia dealbata*	Spain	Methyl cinnamate from flowers	Bioherbicide	[18]
	*A. dealbata*	Spain	Leaf extract	Bioherbicide	[19]
	*A. dealbata*	Portugal	Bark extract	Bioherbicide	[20]
	*A. dealbata*	Spain	Green manure from leaves and small branches	Bioherbicide	[23,24]
	*A. dealbata*	Spain	Bark extract	Stimulating activity	[21]
	*Acacia longifolia*	Spain	Green manure from leaves and small branches	Bioherbicide	[23]
	*Acacia mearnsii*	Not indicated	Leaf and root extracts	Bioherbicide	[22]
	*Acacia melanoxylon*	Not indicated	Flower extract	Bioherbicide	[22]
	*Acacia saligna*	Not indicated	Flower extract	Bioherbicide	[22]
	*Acacia retinodes*	Not indicated	Flower extract	Bioherbicide	[22]
Soil improvers and fertilizers	*Acacia cyclops*	Canada, Marrakech	Shredded branches	Compost, growing substrates	[36,37]
	*A. dealbata*	Spain	Leaves and small branches	Green manure, organic fertilizer	[24]
	*A. dealbata*	Spain	Aerial biomass mixed with sewage sludge	Compost, soil amendment	[33]
	*A. dealbata*	Spain	Leaves, flowers and young branches	Vermicompost	[34]
	*Acacia decurrens*	Ethiopia	Wood	Biochar, soil amendment	[35]
	*A. longifolia*	Portugal	Aerial biomass	Compost, soil amendment	[10]
	*A. longifolia* and *Acacia melanoxylon*	Portugal	*Acacia* aerial biomass	Compost	[29,30]
	*A. longifolia* and *Acacia melanoxylon*	Portugal	*A. longifolia* aerial biomass mixed with pine bark	Compost	[31]
	*A. longifolia*	Portugal	Leaves and small branches mixed with municipal compost	Compost	[32]
	*Acacia mearnsii*	Brazil	Bark bagasse mixed with bovine manure	Vermicompost	[38]
	*Acacia podalyriifolia*	South Africa	Aerial biomass mixed with herbaceous plants	Compost	[28]

## 4. Final Remarks and Considerations

**Figure 1:**
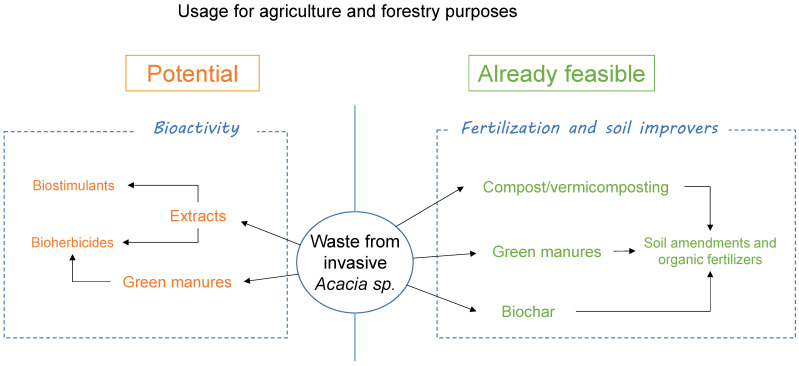
Classification of potential or feasible agricultural and forestry uses of waste biomass of invasive *Acacia* species.

Invasive *Acacia* spp. continue to expand rapidly [39,40]. Current control strategies for *Acacia* invasions are expensive and ineffective on a large scale and in the long term [11]. Therefore, urgent and additional efforts are required to manage invasions of *Acacia*. The considerable amount of biomass waste generated during management actions can be considered as a low-cost resource for obtaining different end-products, and this is receiving increasing interest [7,15,41]. In our opinion, soil agricultural and forestry purposes should be prioritized as a way to sequester the carbon released during this management. We highlight the promising use of *Acacia* waste to produce compost/vermicompost, green manure and charcoal to improve soil fertility and reduce the application of inorganic fertilizer. Nursery and field studies have demonstrated the feasibility of using these products for growing some crops and trees and have proved to be a starting point for evaluating their application in different agricultural and forestry systems (Figure 1). In addition, compost, green manure and charcoal feed on and process large quantities of *Acacia* waste obtained after control actions. Conversely, the bioactivity (mainly as herbicide) of natural compounds from *Acacia* waste, although valuable, is not yet fully developed due to a lack of field experiments (Figure 1). These uses offer promising avenues for controlling the spread of invasive *Acacia* populations by constantly removing their biomass. Although the commercialization and cultivation of live invasive *Acacia* plants are forbidden in several European countries, the use of their waste is not yet regulated and a legal framework should be defined to prevent the spread of propagules [11]. However, due to the critical status of *Acacia* invasion, we propose to complement current control methods with the use of *Acacia* waste as a combined management strategy. This strategy must be implemented exclusively during the vegetative phenological stage (avoiding seeds) and after the waste is dried/dead to limit dispersion risks, which could facilitate a continuous control of the *Acacia* populations. Additionally, there is a lack of cost–benefit analysis of the use of *Acacia* waste to reduce control costs. We strongly recommend that, as emphasized by Ortega et al. [42], profits from using *Acacia* waste should be directed towards reducing costs related to control in the area occupied by these aggressive invaders, preferably within a regulatory framework.

## Data Availability

Not applicable.
